# Independent Effects of a Herbivore’s Bacterial Symbionts on Its Performance and Induced Plant Defences

**DOI:** 10.3390/ijms18010182

**Published:** 2017-01-18

**Authors:** Heike Staudacher, Bernardus C. J. Schimmel, Mart M. Lamers, Nicky Wybouw, Astrid T. Groot, Merijn R. Kant

**Affiliations:** 1 Institute for Biodiversity and Ecosystem Dynamics, University of Amsterdam, Science Park 904, 1098 XH Amsterdam, The Netherlands; hstauda@gmail.com (H.S.); B.C.J.Schimmel@uva.nl (B.C.J.S.); m.lamers@erasmusmc.nl (M.M.L.); N.R.Wybouw@uva.nl (N.W.); A.T.Groot@uva.nl (A.T.G.); 2 Max Planck Institute for Chemical Ecology, Hans-Knöll-Straβe 8, 07745 Jena, Germany

**Keywords:** *Tetranychus urticae*, *Wolbachia*, *Cardinium*, *Spiroplasma*, symbiosis, plant–herbivore interaction, plant defence

## Abstract

It is well known that microbial pathogens and herbivores elicit defence responses in plants. Moreover, microorganisms associated with herbivores, such as bacteria or viruses, can modulate the plant’s response to herbivores. Herbivorous spider mites can harbour different species of bacterial symbionts and exert a broad range of effects on host-plant defences. Hence, we tested the extent to which such symbionts affect the plant’s defences induced by their mite host and assessed if this translates into changes in plant resistance. We assessed the bacterial communities of two strains of the common mite pest *Tetranychus urticae*. We found that these strains harboured distinct symbiotic bacteria and removed these using antibiotics. Subsequently, we tested to which extent mites with and without symbiotic bacteria induce plant defences in terms of phytohormone accumulation and defence gene expression, and assessed mite oviposition and survival as a measure for plant resistance. We observed that the absence/presence of these bacteria altered distinct plant defence parameters and affected mite performance but we did not find indications for a causal link between the two. We argue that although bacteria-related effects on host-induced plant defences may occur, these do not necessarily affect plant resistance concomitantly.

## 1. Introduction

Herbivores face the challenge of having to consume plant material that, because of herbivory, has been under strong selection to be unpalatable. Hence, herbivores have to cope with plant defences ranging from mechanical barriers, such as thorns and trichomes, to the production of poisonous substances [1,2,3]. Moreover, in many cases the nutrient composition of plant material is poor or unbalanced from a herbivore’s perspective or it contains structural molecules that are hard to digest like cellulose or lignin [1,2]. In some cases, herbivores have established symbioses (i.e., the living together of dissimilar species [4]) with microbes, often as mutualistic symbiosis, to promote the utilization of plant material. For instance, some herbivores provide nutrients and shelter to microbes, while in return they make use of the huge metabolic capabilities of these microbes to feed on otherwise unpalatable plants or plant parts, thereby expanding their niche space [5,6,7,8]. 

Plant usage by herbivores may be facilitated either by beneficial bacterial symbionts directly, or indirectly via an interaction between the bacterium and host plant [9,10,11]. For example, direct facilitation may occur via bacteria that upgrade low quality food, by producing essential amino acids or vitamins that the host diet lacks, or by the production of enzymes which enhance the digestion of refractory food sources [5,6,7]. As an example of indirect facilitation, bacteria associated with oral secretions of the Colorado potato beetle (*Leptinotarsa decemlineata*) were shown to alter plant resistance and turn the host plant into better food [12].

Among arthropods, the most prevalent microbial symbionts are so-called reproductive parasites such as *Wolbachia* (Rickettsiales), *Cardinium* (Cytophagales) and *Spiroplasma* (Entomoplasmatales) [13]. Reproductive parasites commonly secure their prevalence in a host population by increasing the proportion of infected females through various mechanisms including cytoplasmic incompatibility, feminization, parthenogenesis or male killing [13,14,15,16]. In most cases, these manipulations by the symbiont are not beneficial to the host, yet very effective for the persistence of the symbiont in a population. This was demonstrated by a recent study in which approximately 40% of all terrestrial arthropod species was estimated to be infected with *Wolbachia* [17]. However, direct beneficial effects of reproductive manipulators on host fitness are thought to mediate their spread within populations as well, especially when manipulation of host reproduction is weak [18,19]. Accordingly, evidence has accumulated that reproductive manipulators, which have long been considered parasites, can benefit their hosts or vectors in various ways [11,20,21]. For instance, reproductive manipulators have been shown to protect their host against parasites, parasitoids, predators and bacterial or viral infections [22,23,24,25,26,27,28,29,30]. Besides protection, *Wolbachia* is known to function as nutritional mutualist in filarial nematodes [31], while some examples also exist for arthropod hosts [32,33,34]. Additionally, infection of arthropods with *Wolbachia* has been associated with the manipulation of plant physiology [35,36,37]; but see [38].

The two-spotted spider mite *Tetranychus urticae* can harbour several (endo)symbiotic bacteria that are known to be reproductive manipulators in mites [39,40,41,42]. However, the infection status of spider mites was shown to vary widely among and within their populations [39,42]. *Tetranychus urticae* is a highly polyphagous pest species found on over 1100 plant species worldwide, including economically important crops like tomato, cucumber, strawberry, bean and cotton [43,44,45]. Plants have evolved a wide array of defences which are organized by the action of several phytohormones in which jasmonic acid (JA) and salicylic acid (SA) are the two central players [46,47]. In general, defence against biotrophic pathogens is orchestrated by SA, while jasmonates, in particular jasmonic acid-isoleucine (JA-Ile), are crucial for defence against herbivores and pathogens with a necrotrophic lifestyle [48,49]. However, spider mites, such as those of the *T. urticae* Santpoort-2 strain, induce these hormones and associated defences simultaneously [50,51,52,53], and in tomato both defence pathways determine the level of resistance against mites [54,55,56,57,58,59]. Recently, we isolated mites from natural *T. urticae* populations and demonstrated that some of them suppressed JA-mediated defences of tomato to uphold a relatively high reproductive performance on this hostile plant species [52]. One of these strains, designated as DeLier-1, was characterized in more detail and shown to significantly suppress JA- as well as SA-mediated defences [52]. Finally, feeding on tomato by mites of strain Santpoort-2 causes the formation of rusty/brown scars on the leaf surface, similar to those described for the Kanzawa mite [60], while DeLier-1 causes white feeding scars [52]. 

Against the background that (endo)symbiotic bacteria can influence host fitness in various ways and may be involved in the manipulation of plant responses, we investigated the bacterial communities that are associated with the DeLier-1 and the Santpoort-2 strain. We found that the DeLier-1 strain contained *Wolbachia* and *Spiroplasma*, while the Santpoort-2 strain harboured *Cardinium* and *Spiroplasma*. We subsequently treated both mite strains with antibiotics to remove these bacteria and tested if bacterial presence was correlated with mite performance and with induced plant responses in tomato (*Solanum lycopersicum*). Our results indicate that the presence of *Wolbachia* had a positive effect on the survival of the DeLier-1 strain, while we did not find an indication that this effect was due to a *Wolbachia*-dependent change in induced plant responses. Therefore, we conclude that bacteria of the DeLier-1 strain likely affect mite survival and induced plant responses independently. In the Santpoort-2 strain the combined presence of *Cardinium* and *Spiroplasma* was negatively correlated with mite fecundity. This result was paralleled by a high expression level of SA-defence marker genes in the host plant when both bacteria were present in the mites. We discuss if the negative correlation of bacterial presence and mite performance could be due to bacteria-induced alteration of plant responses.

## 2. Results

### 2.1. Bacterial Communities in Antibiotics-Treated and Untreated Mite Lines of Tetranychus urticae DeLier-1 and Santpoort-2

Adult female mites, obtained from laboratory populations of either the *T. urticae* Delier-1 or *T. urticae* Santpoort-2 strains [52], harboured different bacterial communities, as was determined by Illumina sequencing of 16S rRNA derived PCR products (Table 1, Figure S1). Most evidently, in the DeLier-1 strain a high percentage of the reads corresponded with the endosymbiotic bacterium *Wolbachia* sp. (W) (Rickettsiaceae), with an average of 30.41% (±13.58 standard deviation, SD). *Wolbachia* was identified in the Santpoort-2 strain as well, albeit at low relative levels with an average of 0.25% (±0.32 SD). In contrast, in the Santpoort-2 strain a high percentage of reads corresponded to the endosymbiotic bacterium *Cardinium* (C) (Bacteroidaceae), with an average of 29.05% (±8.72 SD). *Cardinium* was also found in the DeLier-1 strain, but at low relative levels with an average of 0.0032% (±0.0037 SD). In addition, the same *Spiroplasma* sp. (S) (Spiroplasmataceae) operational taxonomic units (OTU) was present in similar relative amounts in both mite strains: an average of 4.03% (±1.06 SD) in the DeLier-1 strain and 4.42% (±2.67 SD) in the Santpoort-2 strain. Therefore, the DeLier-1 strain was classified as W+S+ and the Santpoort-2 strain as C+S+. 

To assess the effect(s) of the bacteria on mite performance and induced plant responses, mites from both strains were treated with antibiotics (tetracycline). The antibiotics treatments successfully cleared *Wolbachia* from the DeLier-1 strain, as only very few (0–6) reads were detected per line in the W−S+ and W−S− groups (Table 1, Figure S1). *Spiroplasma* was completely removed in the latter group, but was relatively more abundant in the W−S+ group than in the W+S+ group—i.e., on average at 7.98% (±1.71 SD). The antibiotics treatment was also successful in the Santpoort-2 strain (Figure S1). Only a small fraction of the reads (<0.04%) recovered from the tetracycline-treated C−S− group corresponded to *Cardinium* or *Spiroplasma* (Table 1). Moreover, presence of *Wolbachia*, *Spiroplasma* or *Cardinium* was no longer detectable in the antibiotic-treated groups by means of PCR using genus-specific primers on DNA of individual mites (i.e., in the same mites that were used for Illumina sequencing). 

Besides *Wolbachia*, *Cardinium* and *Spiroplasma*, bacteria from the families Enterobacteriaceae (Enterobacteriales) and Pseudomonadaceae (Pseudomonadales) were also present in varying amounts in all groups and sublines of both mite strains (Figure S1). Other bacterial families reached high relative abundances in some of the sublines—e.g., two different Oxalobacteraceae (Burkholderiales), one in line 5 group C−S−, the other in line 6 group C+S+ and in line 2 group W+S+; Sphingobacteriaceae (Sphingobacteriales) in line 2 group W+S+, or Nocardiaceae in line 4 group W−S+. Taken together, this demonstrates that we cleared the mites from *Wolbachia*, *Spiroplasma* or *Cardinium*, but not from all bacteria residing in and/or on the mites. Therefore, we only focused on the consistent presence/absence of *Wolbachia*, *Spiroplasma* or *Cardinium* in all our analyses, to which we refer when we use the terms “bacteria” or “mite-associated bacteria”.

### 2.2. Effects of Wolbachia, Cardinium and Spiroplasma on Spider Mite Performance

**Figure 1:**
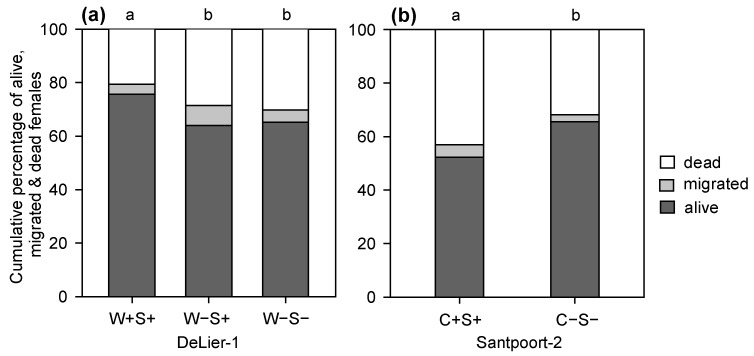
Survival, migration and mortality in two strains of the spider mite *T. urticae* which did (+) or did not (−) contain *Wolbachia* (W), *Spiroplasma* (S) and/or *Cardinium* (C); (**a**) The DeLier-1 strain with three groups: W+S+, W−S+ and W−S−; (**b**) The Santpoort-2 strain with two groups: C+S+ and C−S−. Different letters above the bars indicate significant differences at a level of *p* ≤ 0.05, after applying generalized linear mixed models followed by Tukey multiple comparisons with Holm adjustment. Note that experiments with the Santpoort-2 strain could not be performed with all four lines and that results obtained with the remaining lines were inconsistent, see Figure S2.

**Figure 2:**
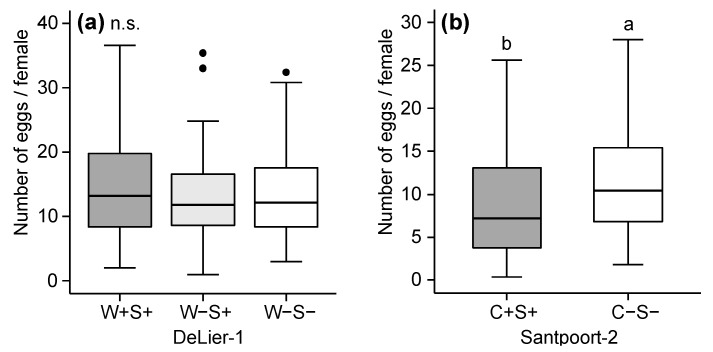
Total number of eggs produced per female in four days in two strains of the spider mite *T. urticae* which did (+) or did not (−) contain *Wolbachia* (W), *Spiroplasma* (S) and/or *Cardinium* (C). (**a**) The DeLier-1 strain with three groups: W+S+, W−S+ and W−S−; (**b**) The Santpoort-2 strain with two groups: C+S+ and C−S−. Boxes span the 25–75 percentiles, horizontal lines in the boxes represent medians, whiskers span 1.5× interquartile range (IQR), dots represent data points outside of this range. Different letters above the boxes indicate significant differences at a level of *p* ≤ 0.05, after applying linear mixed models followed by Tukey multiple comparisons with Holm adjustment. n.s.: not significant.

To assess whether the mite-associated bacteria were correlated with the performance of their host, we determined the number of eggs and survival of adult female mites after four days on tomato plants. For the DeLier-1 strain, we found an overall significant effect of the factor (bacterial) “group” (i.e., presence/absence of *Wolbachia* and/or *Spiroplasma*) on mite survival (Figure 1). More mites of the W+S+ group survived until the end of the experiment compared to the W−S+ and W−S− groups. There was no statistically significant difference in number of eggs between the groups when we calculated the total number of eggs produced per number of females that were initially put on each leaflet (five mites) (Figure 2a).

For the Santpoort-2 strain, overall significantly more mites of the C−S− group survived until the end of the experiment compared to the C+S+ group (Figure 1b). However, this was not consistent among the lines (Figure S2). The number of eggs produced by mites from the C+S+ and C−S− groups was significantly different: overall, mites from the C−S− group produced more eggs than mites from the C+S+ group (Figure 2b). This difference in oviposition did show the same trend for the lines we tested (Figure S3) and this difference was statistically significant for line 5 (*F*_1,29_ = 14.71, *p* < 0.001), but not for line 8 (*F*_1,32_ = 1.21, *p* = 0.27). Notably, the data from the performance assays with the Santpoort-2 strain were not performed with all lines, because line 6 in the C−S− group, as well as line 7 in both groups (C−S− and C+S+), went extinct before the performance assays were completed.

#### 2.2.1. Effects of Mite-Associated *Wolbachia*, *Cardinium* and *Spiroplasma* on Tomato Induced Responses

**Figure 3:**
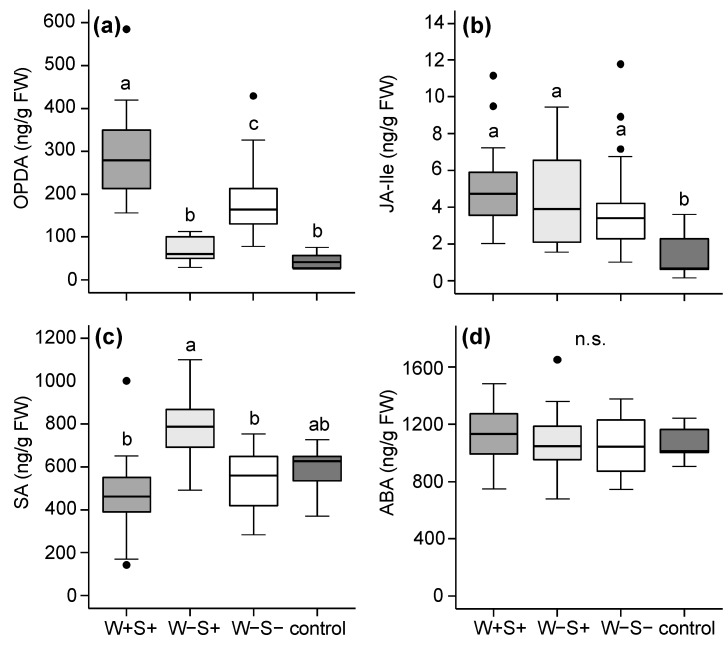
Phytohormone amounts in tomato (*S. lycopersicum*) leaflets after seven days of infestation with the spider mite *T. urticae* DeLier-1 strain which did (+) or did not (−) contain the bacteria *Wolbachia* (W) and *Spiroplasma* (S). Control plants were not infested. Phytohormones for which we assayed include (**a**) 12-oxo-phytodienoic acid (OPDA); (**b**) jasmonic acid-isoleucine (JA-Ile); (**c**) salicylic acid (SA); and (**d**) abscisic acid (ABA). Boxes span the 25–75 percentiles, horizontal lines in the boxes represent medians, whiskers span 1.5× IQR, dots represent data points outside this range. Different letters above the boxes indicate significant differences at a level of *p* ≤ 0.05, after applying linear mixed models followed by Tukey multiple comparisons with Holm adjustment. Phytohormone amounts are presented as nanogram per gram fresh leaf weight (ng·g^−1^·FW). n.s.: not significant.

For the DeLier-1-infested plants, the most prominent differences in phytohormone profiles were found between leaflets infested with mites from the W−S+ group and those infested with the W−S− or W+S+ group (Figure 3). Compared to non-infested control plants, feeding by the W+S+ group resulted in the increased accumulation of 12-oxo-phytodienoic acid (OPDA, a precursor of JA) as did feeding by the W−S− group.

However, leaflets infested with mites from the W−S− group accumulated significantly lower amounts of OPDA than those infested with the W+S+ group. In contrast, the OPDA concentration in leaflets infested with the third group of mites (i.e., the W−S+ group) was similar to that in control leaflets. We did not find this pattern (i.e., lowest hormone concentrations in W−S+ compared to W+S+ and W−S− infested leaflets) for JA-Ile (Figure 3b). The concentration of SA in infested leaflets followed a pattern that appeared opposite of that of OPDA: amounts of SA in leaflets that were infested with the W−S+ group were significantly higher than in leaflets infested with either the W−S− or the W+S+ group, but were not higher than in non-infested control leaflets (Figure 3c). The concentration of abscisic acid (ABA) was not changed after infestations with mites from any of the DeLier-1 groups (Figure 3d). 

**Figure 4:**
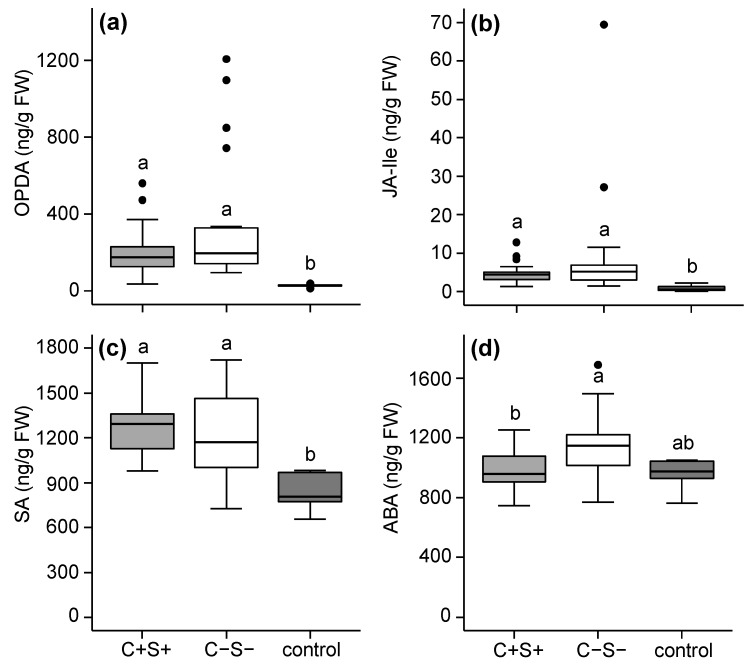
Phytohormone amounts in tomato (*S. lycopersicum*) leaflets after seven days of infestation with the spider mite *T. urticae* Santpoort-2 strain which did (+) or did not (−) contain the bacteria *Cardinium* (C) and *Spiroplasma* (S). Control plants were not infested. Phytohormones for which we assayed include (**a**) OPDA; (**b**) JA-Ile; (**c**) SA; and (**d**) ABA. Boxes span the 25–75 percentiles, horizontal lines in the boxes represent medians, whiskers span 1.5× IQR, dots represent data points outside of this range. Different letters above the boxes indicate significant differences at a level of *p* ≤ 0.05, after applying linear mixed models followed by Tukey multiple comparisons with Holm adjustment. Phytohormone amounts are presented as nanogram per gram fresh leaf weight (ng·g^−1^·FW).

In Santpoort-2-infested leaflets the concentrations of OPDA, JA-Ile and SA were higher after infestation with both the C+S+ and C−S− group compared to uninfested controls, but did not differ between the two groups (Figure 4a–c). Abscisic acid accumulated to higher amounts in C−S− infested leaflets than in C+S+ infested leaflets (Figure 4d).

**Figure 5:**
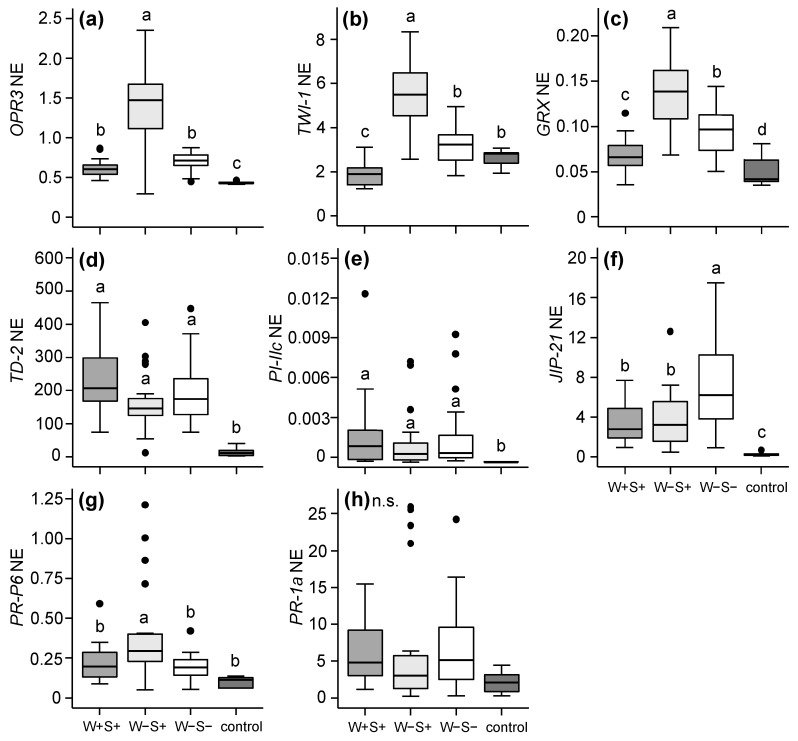
Normalized expression (NE) of plant defence related genes obtained via qRT-PCRs in tomato (*S. lycopersicum*) leaflets after seven days of infestation with the spider mite *T. urticae* DeLier-1 strain which did (+) or did not (−) contain the bacteria *Wolbachia* (W) and *Spiroplasma* (S). Control plants were not infested. (**a**) *OPDA reductase 3* (*OPR3*); (**b**) *Tomato wound-induced 1* (*TWI-1*); (**c**) *Glutaredoxin* (*GRX*); (**d**) *Threonine deaminase-2* (*TD-2*); (**e**) *Proteinase inhibitor IIc* (*PI-IIc*); (**f**) *Jasmonate-inducible protein-21* (*JIP-21*); (**g**) *Pathogenesis-related protein 6* (*PR-P6*); and (**h**) *Pathogenesis-related protein 1a* (*PR-1a*); Gene expression levels were normalized to the levels of tomato *actin*. Boxes span the 25–75 percentiles, horizontal lines in the boxes represent medians, whiskers span 1.5× IQR, dots represent data points outside of this range. Different letters above the boxes indicate significant differences at a level of *p* ≤ 0.05, after applying linear mixed models followed by Tukey multiple comparisons with Holm adjustment. n.s.: not significant.

Previously, it was shown that defence suppression by spider mites, including *T. urticae* DeLier-1, acts downstream of phytohormone accumulation [52]. We therefore augmented the phytohormone data with the expression data of downstream marker genes using quantitative reverse-transcription PCR (qRT-PCR). In the leaflets infested with the DeLier-1 strain, the expression of *OPDA reductase 3* (*OPR3*), which acts directly downstream of OPDA in the JA biosynthesis pathway, was significantly higher in W−S+ than in W+S+ or W−S− infested leaflets (Figure 5a). The same pattern was found for two putative OPDA-responsive genes (ORGs), *tomato wound-induced 1* (*TWI-1*) and *glutaredoxin* (*GRX*) (Figure 5b,c). The expression levels of these ORGs were thus negatively correlated with OPDA amounts and positively with SA amounts, while the coefficient of determination (*R*^2^) values were similar (Figure S4). Expression of the JA-defence marker genes *threonine deaminase-2* (*TD-2*) (Figure 5d), *proteinase inhibitor IIc* (*PI-IIc*) (Figure 5e) and *jasmonate-inducible protein-21* (*JIP-21*) (Figure 5f) was induced by mites from all three groups compared to the control, but for *TD-2* and *PI-IIc* the expression level did not differ among the groups. Transcripts of *JIP-21* accumulated to significantly higher levels in W−S− infested than in W+S+ and W−S+ leaflets (Figure 5f). The expression of the SA-defence marker genes, *pathogenesis-related protein 6* (*PR-P6*) (Figure 5g) and *pathogenesis-related protein 1a* (*PR-1a*) (Figure 5h), was not significantly higher in DeLier-1-infested leaflets than in non-infested controls, irrespective of the bacterial infection-status of the mites. The only exception was the induction of *PR-P6* in W−S+ infested leaflets. It has to be noted that this increased *PR-P6* expression was visually clear in only two out of the four lines we tested (Figure S5). Accordingly, SA amounts showed a moderate but significant correlation with expression levels of *PR-P6* (Figure S6a) and did not correlate with *PR-1a* transcript levels (Figure S6b).

**Figure 6:**
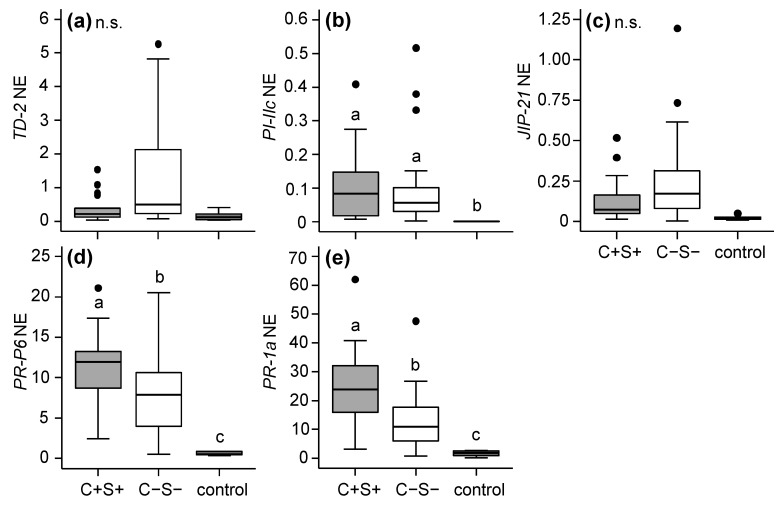
Normalized expression (NE) of plant defence related genes obtained via qRT-PCRs in tomato (*S. lycopersicum*) leaflets after seven days of infestation with the spider mite *T. urticae* Santpoort-2 strain which did (+) or did not (−) contain the bacteria *Cardinium* (C) and *Spiroplasma* (S). Control plants were not infested. (**a**) *TD-2*; (**b**) *PI-IIc*; (**c**) *JIP-21*; (**d**) *PR-P6*; and (**e**) *PR-1a*; Gene expression levels were normalized to the levels of tomato *actin*. Boxes span the 25–75 percentiles, horizontal lines in the boxes represent medians, whiskers span 1.5× IQR, dots represent data points outside of this range. Different letters above the boxes indicate significant differences at a level of *p* ≤ 0.05, after applying linear mixed models followed by Tukey multiple comparisons with Holm adjustment. n.s.: not significant

In the leaflets infested with the Santpoort-2 strain, none of the JA-defence marker genes (*TD-2*, *PI-IIc* and *JIP-21*) were differentially expressed (*p* ≤ 0.05) between C+S+ and C−S− infested leaflets (Figure 6a–c). Interestingly, transcripts of both SA-defence marker genes (*PR-P6* and *PR-1a*) were more abundant in C+S+ infested leaflets compared to C−S− infested leaflets (Figure 6d–e). 

#### 2.2.2. Effects of *Wolbachia*, *Cardinium* and *Spiroplasma* on the Amount of Feeding Damage Inflicted by Spider Mites

**Figure 7:**
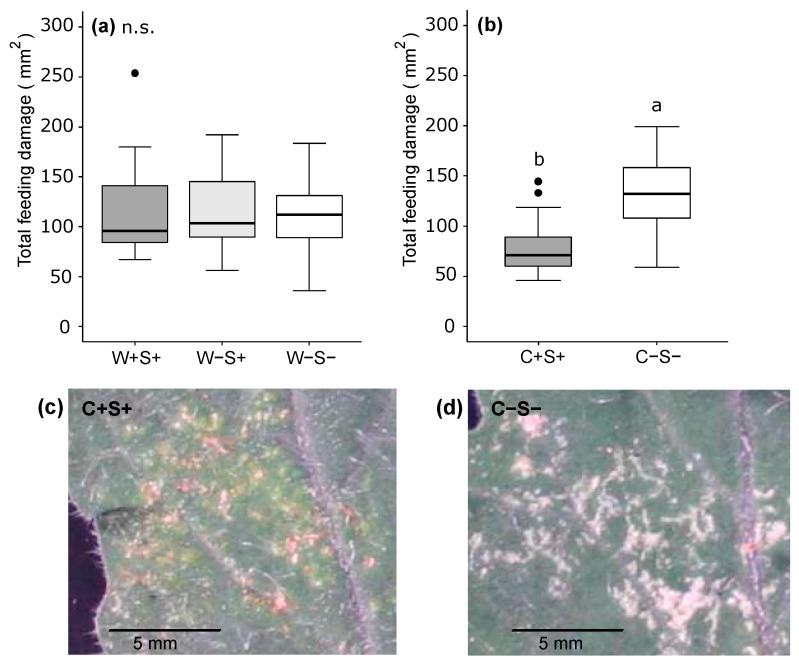
Feeding damage on tomato (*S. lycopersicum*) leaflets inflicted by two strains of the spider mite *T. urticae* which did (+) or did not (−) contain the bacteria *Wolbachia* (W), *Spiroplasma* (S) and/or *Cardinium* (C). Total amount of feeding damage (mm^2^) after seven days of infestation by mites from (**a**) the DeLier-1 strain with three groups: W+S+, W−S+ and W−S−; and (**b**) the Santpoort-2 strain with two groups: C+S+ and C−S−. Boxes span the 25–75 percentiles, horizontal lines in the boxes represent medians, whiskers span 1.5× IQR, dots represent data points outside of this range. Different letters above the boxes indicate significant differences at a level of *p* ≤ 0.05, n.s.: not significant; (**c**) Typical rusty red/brown scars inflicted by feeding of the C+S+ group of the Santpoort-2 strain and (**d**) typical white scars inflicted by feeding of the C−S− group of the Santpoort-2 strain.

To test if the magnitude of induced plant responses was correlated with the amount of feeding by the mites, we quantified the spider mite-inflicted feeding damage (recognizable as chlorotic spots) on the same leaflets that were used for phytohormone extractions and tomato RNA isolation for qRT-PCRs. Overall, there was no significant difference in feeding damage between the three groups (W+S+, W−S+, W−S−) of the DeLier-1 strain (Figure 7a). In contrast, there was a clear difference in the amount of feeding damage caused by the two groups of the Santpoort-2 strain and in the type of damage these inflicted. Not only did mites from the C−S− group feed significantly more than mites from the C+S+ group (Figure 7b), feeding by the C+S+ group resulted in rusty red/brown “scars” on the leaflets (Figure 7c), while those infested with the C−S− group had clear white scars (Figure 7d).

## 3. Discussion

**Figure 8:**
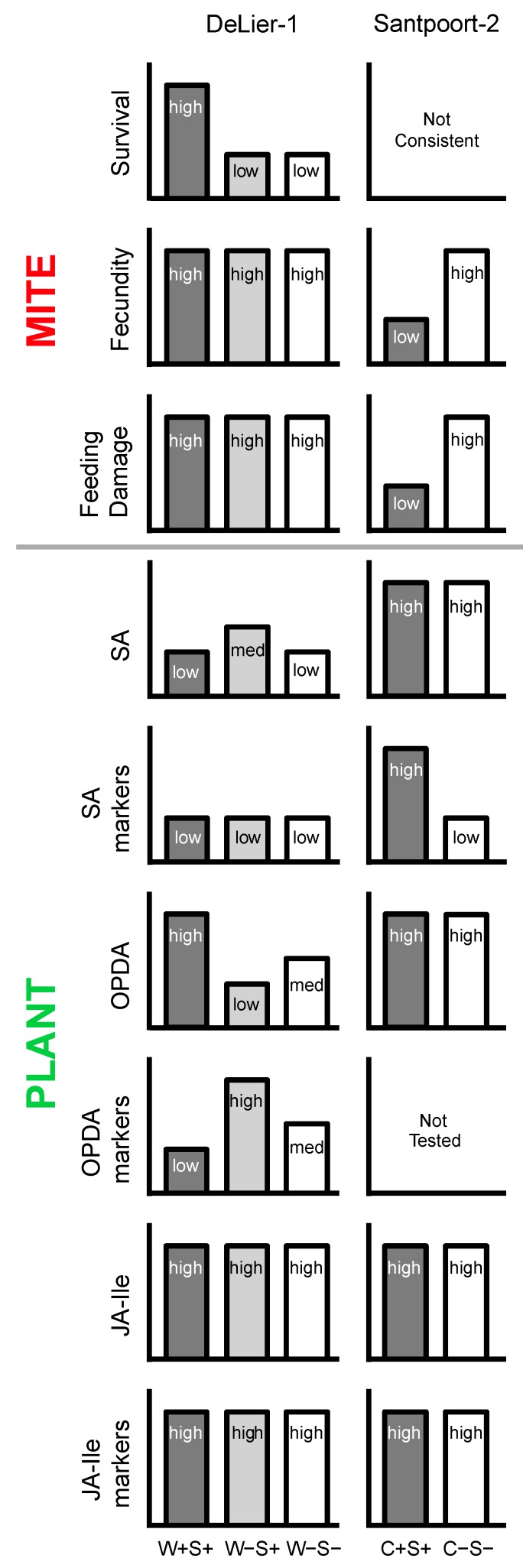
Schematic and simplified overview of the most important findings of this study. Adult female spider mites (*T. urticae*) from the DeLier-1 strain and the Santpoort-2 strain were treated with antibiotics to remove their associated bacteria *Wolbachia* sp. (W), *Spiroplasma* sp. (S) and/or *Cardinium* (C), after which the indicated mite and plant (tomato; *S. lycopersicum*) parameters were assayed. For a more detailed description we refer to the Results and Discussion sections. med: intermediate; +: bacteria present; −: bacteria absent.

In this study we investigated two strains of *T. urticae* that differed in their bacterial symbionts. The DeLier-1 strain, contains *Wolbachia* (W) and *Spiroplasma* (S) bacteria, while the Santpoort-2 strain harbours *Cardinium* (C) and *Spiroplasma* (i.e., the same OTU as in the Delier-1 strain)*.* Using antibiotic treatments we removed these well-known reproductive parasites to determine their effect(s) on spider mite performance and on induced plant responses. We showed that: (i) the presence of the (endo)symbionts correlated positively with the survival, but not with number of eggs produced by the DeLier-1 strain, while it correlated negatively with the number of eggs produced by the Santpoort-2 strain; (ii) Plant responses to mite infestations differed between the mite groups that did or did not harbour *Wolbachia*, *Cardinium* and/or *Spiroplasma*; (iii) The *combined* presence of *Wolbachia* and *Spiroplasma* bacteria had consequences for induced plant responses, indicating that these bacteria might interact. See Figure 8 for a schematic overview of the results.

It has to be noted that, although the antibiotic treatments resulted in the (near) complete removal of *Wolbachia*, *Cardinium* and/or *Spiroplasma* from the mites (Table 1), various other bacterial strains were present in or on antibiotics-treated and untreated mites as well (Figure S1). These bacterial strains were not consistently present in all the mite lines of the same group and are thus not likely to be responsible for the differences that we found between the groups (but see below for the discussion on the performance of mites from the Santpoort-2 groups). In addition, tetracycline treatments may have had effects on the mites other than the removal of bacteria. Direct toxic effects of tetracycline, such as inhibition of mitochondrial functioning, are unlikely to play a role in our study, because we started experiments at least 15 generations after the antibiotics treatments. However, we cannot rule out that the tetracycline treatment had selective effects, such as the selection for more toxin resistant mites. 

### 3.1. Effects of Wolbachia, Spiroplasma and Cardinium on Spider Mite Performance

When we investigated the performance of the DeLier-1 strain in a four-day trial, survival was highest in mites containing *Wolbachia* and *Spiroplasma*. The presence or absence of *Spiroplasma* did not seem to affect mite survival, as the survival rate was similar for the W−S+ and W−S− groups. We did not find more spider mite eggs on leaflets infested with the W+S+ group than on those infested with the W−S+ or W−S− group. However, since differences in mite survival can be expected to have a significant effect on reproductive performance in the long run, future experiments should focus on the lifetime production of eggs by the DeLier-1 strain with and without symbionts.

For the Santpoort-2 strain, egg production was negatively correlated with the presence of *Cardinium* and *Spiroplasma*. However, the performance assay for this mite strain could not be carried out for all lines (see Material and Methods section), hence we have to be cautious with drawing conclusions about the effect of *Cardinium* and *Spiroplasma* on the fitness of their Santpoort-2 hosts. For instance, the number of remaining lines was insufficient to firmly test whether these symbionts affect the survival of their host, because unlike for egg production, mites from the remaining lines showed inconsistent survival patterns. Moreover, both survival and egg production in these remaining lines (also) coincide with the presence of bacteria other than *Cardinium* and *Spiroplasma* (i.e., Oxalobacteriaceae).

Previous studies indicate that the effect of reproductive parasites on the fitness of their host may strongly depend on the genotype of both symbiont and host and whether or not symbionts optimize their prevalence in a population by reproductive manipulation [19,21,62]. Accordingly, the reported effects of *Cardinium*, *Wolbachia* and *Spiroplasma* on the fitness of their arthropod host, including other *T. urticae* strains than the ones tested here, are diverse (i.e., positive, negative or no effects) [42,63,64,65,66,67,68,69,70,71,72,73,74]. 

### 3.2. Effects of Wolbachia, Cardinium and Spiroplasma on Tomato Induced Responses

When we investigated the effect of *Wolbachia* and *Spiroplasma* infection of the DeLier-1 strain on tomato induced responses, the most striking finding was that OPDA amounts did not significantly increase in W−S+ infested leaflets compared to non-infested leaflets, while the OPDA concentration was highest in W+S+ infested leaves and intermediate in W−S− infested ones. Thus, the presence of *Wolbachia* in the DeLier-1 strain was correlated with enhanced OPDA accumulation, while the presence of *Spiroplasma* in these mites was correlated with suppression of OPDA accumulation. Expression of *OPR3* was highest in W−S+ infested leaflets suggesting that the conversion rate of OPDA might have been increased and thereby have reduced OPDA accumulation in the respective leaflets. However, the end product of the oxylipin pathway, JA-Ile, which is considered the main biologically active jasmonate [75,76], did not show any pattern that was correlated with bacterial presence. It remains to be investigated why the accumulation of OPDA is affected by spider mite-associated bacteria, while that of JA-Ile is not. In addition, it is currently unknown if OPDA plays a JA-independent role in resistance against *T. urticae*. It has been demonstrated that OPDA can confer plant resistance to several herbivore and pathogen species, i.e., via inducing plant responses independent of JA-Ile biosynthesis and/or signalling, and via its cytotoxic properties [77,78,79,80,81,82,83,84,85,86]. However, in our study the detected OPDA accumulation pattern was not reflected in the mite performance data.

In *Arabidopsis*, OPDA specifically induces the expression of *uridine diphosphate-glycosyltransferase 73B5* (*UGT73B5*) and *GRX480* [78,82,87]. Unexpectedly, in DeLier-1-infested leaflets the expression levels of the tomato homologs of these ORGs, *TWI-1* and *GRX*, respectively, correlated negatively with OPDA amounts, but correlated positively with SA amounts. Tomato *TWI-1* and *GRX* may simply not be good OPDA markers, which emphasizes that it can be risky to rely on sequence similarity for predicting functional or regulatory similarities. Furthermore, considering the positive correlation with SA, *TW-1* and *GRX* may be regulated by SA rather than by OPDA. Interestingly, also some *Arabidopsis* ORGs, including *UGT73B5* and *GRX480*, have been shown to be SA-responsive [88,89,90]. Tomato *TWI-1* is also responsive to SA [91], for *GRX* this is not known yet.

Salicylic acid concentrations correlated only weakly with expression levels of *PR-P6* and did not correlate with *PR-1a* expression levels at all in DeLier-1-infested leaflets. The expression of *PR-P6* is induced upon exogenous application of SA or its synthetic analogue benzothiadiazole BTH [92,93] and the *PR-P6* promoter has been demonstrated to be responsive to SA [94]. Likewise, the expression of *PR-1a* is induced upon exogenous application of BTH [95]. A tomato infestation with defence-inducing *T. urticae* Santpoort-2 (i.e., untreated C+S+ group) also results in the increased accumulation of SA as well as transcripts of *PR-P6* and *PR-1a* (Figure 4c and Figure 5g,h, respectively, and [52]). Importantly, the induced SA-mediated defence response was found to have a negative effect on the performance of the Santpoort-2 mites [59]. The weak or lack of correlation between SA levels and *PR* gene expression in DeLier-1-infested leaflets possibly resulted from defence suppression by the mites, which has been shown to occur downstream of SA (and JA) accumulation (e.g., at the *PR-1a* transcript level) [52]. It is also possible that the induction of *PR* genes by exogenous application of (high concentrations of) SA or BTH is more effective than endogenous SA accumulation and *PR* gene expression upon herbivory since the latter is subject to spatio-temporal dynamics of multiple hormonal responses. The usefulness of such marker genes as predictors of SA responses deserves further testing in future experiments. 

Together, our findings for the DeLier-1 strain indicate that bacteria affected the measured plant responses and mite survival independently. We did not find indications that bacteria affect plant resistance against mites of the DeLier-1 strain. Although plant defence responses mediated by jasmonates and SA appear to be most important to confer resistance to *T. urticae* [50,51,53,54,55,56,57,58,59], we cannot exclude that mite-associated bacteria affected other plant responses (e.g., those regulated by ethylene) or other host fitness parameters than we have surveyed. Note that only very few ethylene response-associated genes were found to be differentially expressed in tomato upon spider mite infestation [50,53]. Furthermore, the altered plant responses that we found in DeLier-1-infested leaves cannot be explained by the amount of damage inflicted due to mite feeding, as this was equal among all three groups. 

When investigating the Santpoort-2 strain, we found that leaflets infested with mites from the C−S− group contained higher amounts of ABA than C+S+ infested leaflets, while transcript levels of SA-responsive *PR-P6* and *PR-1a* were reduced. Consistent with these results, earlier studies found that feeding by arthropods with certain bacterial symbionts was associated with increased amounts of SA and higher expression levels of *PR* genes in host plants [12,96]. However, in our study accumulation of the phytohormone SA itself was not affected by bacterial presence. One explanation for the altered expression of the *PR* genes could be negative crosstalk between ABA and SA signalling pathways, which has been described in *Arabidopsis* [97,98]. Also in tomato, ABA appears to negatively regulate SA defences, in particular by inhibiting expression of *PR-1a* [99]. The reduced expression of *PR* genes in C−S− samples compared to the C+S+ samples might thus be explained by the negative action of ABA on these genes. Notably, mites from the C−S− group caused almost twice as much feeding damage as mites from the C+S+ group did, suggesting that mites from the C−S− group fed more while JA and SA defence responses were induced to a similar, or even lower (*PR* genes), magnitude. The exact role of the PR proteins in defence against spider mites remains to be investigated.

Our finding that induced plant responses are affected by bacterial presence in the Santpoort-2 strain was complemented with different feeding scar phenotypes of C−S− and C+S+ mites on tomato leaflets. Whereas feeding by the C+S+ group resulted in rusty red/brown scars, infestation with the C−S− group yielded white scars. Similar scar phenotypes have been reported before from the Kanzawa spider mite *Tetranychus kanzawai*. In that case, red scars were associated with increased SA amounts of bean leaves as well as increased expression of a SA marker gene [100]. In the Kanzawa spider mite, mite genotype and not maternally inherited symbionts were the cause of differentially coloured scars upon *T. kanzawai* feeding [60]. Nevertheless, since the genetic background of our mite lines was equal among the C+S+ and C−S− groups, we suggest that the red scars in our case did probably not have a genetic basis but may have been caused by the presence of a bacterium. Most likely the presence of *Cardinium* resulted in rusty red/brown scars, because we did not observe this scar phenotype with mites from the W−S+ group of the DeLier-1 strain that contained the same *Spiroplasma* OTU.

### 3.3. The Combined Presence of Wolbachia and Spiroplasma Bacteria Has Consequences for Induced Plant Responses

Within a host, the various symbionts that (can) co-occur possibly interact, which may affect host and bacterial fitness in various ways. Our results suggest that a single or double infection status of the DeLier-1 suppressor strain differentially affects plant defence responses but not mite performance (and thus probably not plant resistance). In these mites, the presence of *Spiroplasma* partially antagonized *Wolbachia*-associated changes in plant responses. This could have resulted from independent effects of the bacteria on mites and/or plant responses. Alternatively, bacteria may interact in the mites and competition between the two symbionts, for instance for space and/or resources, might underlie our findings. Microbial competition for space (i.e., the ovaries) within the mosquito hosts *Anopheles stephensi* and *Aedes aegypti* was reported to occur between *Wolbachia* and an unrelated *Asaia* bacterium [101,102]. Besides the proper localization, symbionts may reduce densities of a second symbiont in a host. Density is an important parameter for fidelity of vertical transmission of bacteria as well as for their effect on host fitness [103,104,105,106,107]. Our Illumina MiSeq analysis showed that the relative abundance (as well as the total number of reads) of *Spiroplasma* in the lines of the W−S+ group was higher than in the lines of the W+S+ group, suggesting that *Wolbachia* may negatively affect the abundance of *Spiroplasma* in the DeLier-1 strain. However, since 16S amplicon sequencing is only a semi-quantitative method, bacterial abundance should be assessed by means of qRT-PCRs to test this hypothesis. 

Unfortunately, we did not obtain all bacterial combinations in *T. urticae* to disentangle the exact effects of single bacterial strains and their combined effects on mite fitness and induced plant responses. For instance, we did not have mites that were infected with only *Wolbachia*, hence we can only indirectly infer the role of *Wolbachia* in our experiments from comparisons between the W+S+, W−S+, and W−S− groups. Moreover, for the Santpoort-2 strain the roles of *Spiroplasma* and *Cardinium* remain difficult to interpret because we did not have C−S+ or C+S− groups. The same *Spiroplasma* OTU occurred in both the Santpoort-2 inducer and the DeLier-1 suppressor strain. If we assume that *Spiroplasma* in the Santpoort-2 strain had the same effect on host fitness parameters as in the DeLier-1 strain, we could attribute the negative effects of bacteria in the Santpoort-2 strain to *Cardinium*. However, *Spiroplasma* may interact with *Cardinium* in the mites. Furthermore, its effects on host fitness may depend on mite genotype [108].

## 4. Material and Methods

### 4.1. Plants

Tomato (*S. lycopersicum* cv. Castlemart) and bean (*Phaseolus vulgaris* cv. Speedy) were germinated and grown in a greenhouse (25/18 °C day/night temperature, 16 light (L)/8 dark (D) photoperiod, 50%–60% relative humidity). Experiments involving plants were carried out in a climate room (default settings: 25 °C, 16L/8D photoperiod, 60% relative humidity, 300 µE·m^−2^·s^−1^), to which plants were transferred seven days in advance.

### 4.2. Spider Mites

We used spider mites from the Santpoort-2 strain and the DeLier-1 strain. The Santpoort-2 strain has been described before as inducer of tomato JA and SA defences, to which this strain is also susceptible [52,59], while the DeLier-1 strain was shown to suppress some of these defences [52]. Spider mites from both strains were reared separately on detached bean leaflets in a climate room.

#### 4.2.1. Bacterial Communities in Antibiotics-Treated and Untreated Mite Lines of *Tetranychus urticae* DeLier-1 and Santpoort-2

Results from a preliminary assessment of the presence of bacteria in the two mite strains indicated that they harboured different endosymbiotic bacteria. The DeLier-1 strain contained *Wolbachia* sp., while *Cardinium* was identified in the Santpoort-2 strain. In addition, *Spiroplasma* sp. was found in both mite strains (see also Table 1, Figure S1).

#### 4.2.2. Antibiotics Treatments and Nomenclature of Mite Lines

We treated mites from both strains with antibiotics to remove *Wolbachia*, *Cardinium* and *Spiroplasma* bacteria. Offspring from randomly selected mated adult females (from laboratory mass rearings) was divided over two treatments: (i) antibiotics-treated; and (ii) untreated controls. For the antibiotics treatment, adult female spider mites were first kept on bean leaf discs on cotton wool soaked with tetracycline hydrochloride (Sigma-Aldrich, St. Louis, MO, USA) for 2–3 days. After this, they were transferred to new leaf discs on water-saturated cotton wool to produce eggs. Two days later, adult females were individually sampled in Eppendorf tubes and stored at −80 °C until DNA was extracted for diagnostic PCRs to test the bacterial infection status of the mites (see below). The eggs on the leaf discs were allowed to hatch and mature in a climate room, after which the antibiotics treatment was repeated. In parallel, untreated control mites were kept on leaf discs placed on water-saturated cotton wool. After egg production, mites were sampled for diagnostic PCRs as described below. Three subsequent generations of mites were treated in this way, but with increasing concentrations of tetracycline (i.e., 0.15%, 0.20% and 0.30% *w*/*v*) to obtain mites free of *Wolbachia*, *Cardinium* and *Spiroplasma* as assessed via diagnostic PCR. From generation 4 onwards, all mites were reared on untreated detached bean leaflets to accommodate larger populations. 

During the antibiotics treatment, we kept track of the individual mites and their offspring and only kept those “lines” (antibiotics-treated versus control group) that both originated from the same adult female (i.e., these were “sister lines”). This was done to minimize genetic variation between antibiotics-treated and untreated control groups. Following these criteria, we obtained four lines for the DeLier-1 strain, designated as line 1, 2, 3 and 4. Each of the four lines had three sublines: W+S+ contained both *Wolbachia* and *Spiroplasma*; W−S+ was free of *Wolbachia*, but contained *Spiroplasma*, W−S− was free of *Wolbachia* and *Spiroplasma*. We did not obtain W+S- sublines. For the Santpoort-2 strain, we obtained four lines as well, which were designated as lines 5, 6, 7 and 8. Each of the four lines had two sublines: C+S+ contained *Cardinium* and *Spiroplasma*, and C−S− was free of *Cardinium* and *Spiroplasma*. Sublines with the same respective bacteria will be referred to as “groups”. For the DeLier-1 strain the groups were W+S+, W−S+ and W−S−, for the Santpoort-2 strain the groups were C+S+ and C−S− (see Table 1, Figure S1 for an overview of the mite lines). Mites from each strain and subline were regularly checked for their bacterial infection status by diagnostic PCR and kept on untreated detached bean leaflets for at least 15 generations before they were used for the plant infestation assay and mite fecundity tests.

#### 4.2.3. Illumina Sequencing

To assess the presence of *Wolbachia*, *Cardinium*, *Spiroplasma* and other potentially present bacteria in mites from the five groups (W−S−, W−S+, W+S+, C−S− and C+S+) that were used for the plant infestation assay, we sampled five tomato-habituated mites per subline (as described above) for Illumina sequencing. DNA was extracted from single mites using a fast Chelex method modified from [39]. To isolate the DNA, a single mite was ground and homogenized in 100 μL sterile 5% *w*/*v* Chelex (Sigma-Aldrich) with a sterile pestle, after which 2.5 μL proteinase K (20 mg/mL, Sigma-Aldrich) was added. Samples were then incubated at 56 °C for 1 h, followed by incubation at 95 °C for 8 min to complete the DNA extraction. DNA from the five mites from the same subline was pooled to form one sample. DNA concentration was adjusted to 25–35 ng/μL per sample. In total, 20 (pooled) samples were sent for sequencing, one for each subline. Amplification and sequencing of the 16S rRNA gene fragment was done by LGC Genomics (Berlin, Germany) using an Illumina MiSeq sequencer (2 × 250 bp paired-end reads; Illumina, San Diego, CA, USA) and the universal primers 341F and 785R modified from [61], see Table S1. Since the Chelex method does not yield highly pure DNA, the initial PCR amplification of the 16S rRNA genetic region was done on 20 times diluted DNA. Furthermore, the PCR was run with 35 instead of the usual 30 cycles.

Sequences were provided as adapter clipped FASTQ files and analysed in Quantitative Insights into Microbial Ecology (QIIME), which is a standard pipeline for microbial community analysis [109]. First, forward and reverse reads were joined with the join_paired_ends.py algorithm. Joined sequences were quality filtered, applying a Phred threshold of 20. Subsequently, sequences were clustered into OTUs with the open reference OTU picking command, applying the uclust algorithm [110] and 97% similarity cut-offs. First, sequences were clustered against the reference Greengenes 16S rRNA gene database [111]. Sequences that did not cluster with the reference sequences were clustered de novo. The most abundant sequence from each OTU cluster was taken as representative sequence. Representative sequences were aligned with PyNAST, using the Greengenes core set as a template [112]. PyNAST-aligned sequences were checked for chimeras with Chimera Slayer [113]. Identified chimeras were removed from de novo clustered sequences for downstream analysis. Taxonomy was assigned to the representative sequences using the uclust consensus taxonomy classifier [110]. The resulting OTU table was manually edited; global singletons and sequences identified as chloroplast and mitochondrial DNA were removed from the dataset. In Figure S1, we show OTUs that were present above 0.5% in at least one of the sublines.

#### 4.2.4. Diagnostic PCRs on Mites

To verify the presence or absence of the most common endosymbionts in mites (i.e., *Wolbachia*, *Cardinium* and *Spiroplasma* [39,40,41,42]), we performed diagnostic PCRs on DNA extracted from spider mites using genus-specific bacterial primers (Table S1). Adult female mites were sampled in Eppendorf tubes and their DNA was extracted using the Chelex method as described above. Samples were stored at 4 °C until they were used for PCR as previously described [114].

### 4.3. Effects of Wolbachia, Cardinium and Spiroplasma on Spider Mite Performance

#### 4.3.1. Spider Mite Performance Assay

To establish whether (endo)symbionts had an effect on mite performance, we assessed spider mite fecundity and survival on wild type tomato plants. For the experiment, an “egg-wave” [52] was generated by allowing random adult females from each strain to produce eggs on the adaxial surface of detached bean leaflets, which had been put flat on wet cotton wool. After 48 h of egg production, all mites were removed from the leaflets, collected in Eppendorf tubes (25–35 mites from the same subline were pooled), flash-frozen in liquid nitrogen and stored at −80 °C until their DNA was extracted for diagnostic PCRs. The eggs were allowed to hatch and mature in a climate room for another nine days. The bean leaflets with mites were then transferred to leaves of 21-day-old tomato plants to habituate the mites to tomato to minimize possible effects of the previous diet (i.e., bean) on mite behaviour, performance, and/or induced tomato responses. Dietary effects are known to persist for at least 48 h, after which they diminish rapidly [115]. Three days later, the 1 ± 1-day-old adult female mites were collected from the tomato leaves and transferred to new 21-day-old tomato plants for the mite performance assay. Plants were infested with five mites per leaflet; three leaflets per plant; 3–6 plants per treatment. A lanolin (Sigma-Aldrich) barrier was made around the petiolule to prevent the mites from escaping. After four days, the number of eggs produced by the mites, as well as the number of alive, dead and missing (i.e., migrated) mites, was recorded using a stereo microscope (Leica MZ6; Leica Microsystems, Wetzlar, Germany). This experiment was repeated 2–3 times for all four lines of the DeLier-1 strain. However, for the Santpoort-2 strain, populations from the C−S− subline of line 6, as well as both sublines of line 7 went extinct (for yet unknown reasons) before we could complete the performance assays. Data from the C+S+ subline of line 6 was included for analysis.

#### 4.3.2. Statistical Analysis of Spider Mite Performance Assay

To test the effect of *Wolbachia*, *Cardinium* and/or *Spiroplasma* on mite oviposition, we constructed one linear mixed effect model (LMM) for each mite strain (DeLier-1 and Santpoort-2) in the lme4 package [116], using “average number of eggs per number of females that were originally put on the leaves” (five mites) as response variable. To test effects of bacteria on survival of the mites, we used generalized linear mixed models (GLMMs) in the lme4 package using a binomial distribution to analyze the proportion of mites that were dead or alive at the end of the oviposition experiment (i.e., after four days). In the models of oviposition and survival, we used (bacterial) “group” as explanatory variable and “line” was added as random effect. Additionally, since experiments were spread over different experimental days, and in total 6–9 plants were used per line (with three leaflets per plant), we added a nested random effect with “leaflet” nested in “plant”, nested in “day” (1|day/plant/leaflet) to the model. Pairwise comparisons for the DeLier-1 strain were done using Tukey contrasts in the multicomp package [117] and applying Holm adjustments to account for multiple comparisons. The response variables were transformed using log, sqrt or 1/sqrt if necessary for meeting the assumptions of homogeneity of variance. All GLMMs were checked for overdispersion by taking the sum of Pearson residuals squared and dividing by the residual degrees of freedom. The GLMMs were found to be not overdispersed. All analyses were performed using the statistical software R 3.0.2 [118].

### 4.4. Effects of SpiderMite-Associated Wolbachia, Cardinium and Spiroplasma on Induced Plant Responses

#### 4.4.1. Plant Infestation Assay

To measure phytohormone levels and plant defence gene expression upon mite-inflicted feeding damage, tomato plants were infested with spider mites as described previously [52], but prior to the infestation, mites were habituated on tomato for two days. For the experiment, we used adult female spider mites of similar age, obtained from an egg-wave as described above. After removal of the females, eggs were allowed to hatch and mature in a climate room for another 12 days. The bean leaflets with mites were then taken from the cotton wool and placed upside-down on leaves of 28-day-old tomato plants to infest these (i.e., habituation step). Two days later, the 3 ± 1-day-old adult female mites were collected and transferred to 21-day-old tomato plants for the plant infestation assay, according to our standard infestation protocol; 15 mites per leaflet, three leaflets per plant. To prevent the mites from escaping, a lanolin (Sigma-Aldrich) barrier was made around the petiolule, which was also applied to uninfested control leaflets. A total of five plants were infested per mite subline (i.e., 20 plants per group). To verify the bacterial infection status of each strain, five tomato-habituated mites per strain were individually collected in Eppendorf tubes, flash-frozen in liquid nitrogen and stored at −80 °C until DNA was extracted for PCR amplification and subsequent Illumina sequencing of the 16S rRNA genetic region (see “Illumina sequencing”, Section 4.2.3). 

At seven days post-infestation, mites and tomato leaflets were harvested separately. First, spider mites were removed from the leaflets with a vacuum pump, a sterile 1 ml pipet tip, and mite-proof gauze to quickly remove the mites without touching and hence mechanically damaging the leaflets. Subsequently, the mite-cleared leaflets were excised without the petiolule. The three detached leaflets obtained from the same plant, along with a scale marker, were then aligned on black paper, gently covered with a thin glass plate to flatten them out, and photographed with a Canon EOS 300D DSLR camera (Canon, Tokyo, Japan) equipped with a Canon EF-S 18-55 mm lens to enable in silico calculation of spider mite-inflicted feeding damage, using Adobe Photoshop CS6 Extended (Adobe Systems, San Jose, CA, USA) as described by [50]. Finally, the leaflets were flash-frozen in liquid nitrogen and stored at −80 °C until we extracted their phytohormones and RNA. The three leaflets obtained from the same plant were pooled to form one biological replicate. In total, it took less than 5 min per plant to complete these three steps and harvest the leaflets. Care was taken to not damage them. Except for removal of the mites, non-infested control leaflets were processed in the same way. 

#### 4.4.2. Isolation of Phytohormones and Analysis by Means of Liquid Chromatography Tandem Mass Spectrometry 

Per sample, 200–300 mg of frozen leaf material was homogenized (Precellys 24; Bertin Technologies, Aix-en-Provence, France) in 1 mL of ethyl acetate which had been spiked with D_6_-SA and D_5_-JA (C/D/N Isotopes Inc., Pointe-Claire, QC, Canada) as internal standards with a final concentration of 100 ng·mL^−1^. Tubes were centrifuged at 13,000 rpm (15,493× *g*; Sigma 3-30KS; SIGMA Laborzentrifugen GmbH, Osterode am Harz, Germany) for 10 min at 4 °C and the supernatant (the ethyl acetate phase) was transferred to new tubes. The pellet was re-extracted with 0.5 mL of ethyl acetate (without internal standards) and centrifuged again at 13,000 rpm for 10 min at 4 °C. Both supernatants were combined and then evaporated to dryness on a vacuum concentrator (CentriVap centrifugal concentrator; Labconco, Kansas City, MO, USA) at 30 °C. The residue was re-suspended in 0.1 mL of 70% methanol (*v*/*v*), centrifuged at 14,800 rpm (20,081× *g*) for 15 min at 4 °C, and the supernatants were transferred to glass vials and then analysed using a liquid chromatography tandem mass spectrometry (LC–MS/MS) system (Varian 320-MS LC/MS; Agilent Technologies, Santa Clara, CA, USA). A serial dilution of pure standards of ABA, OPDA, JA, JA-Ile and SA was run separately. We injected 10 µL of each sample onto a Kinetix 5u C18 100A column (C18 phase, 5 μm particle size, 100 Å pore size, 50 × 2.1 mm; Phenomenex, Torrance, CA, USA) equipped with a Phenex-RC guard cartridge (Phenomenex). The mobile phase comprised of solvent A (0.05% formic acid in LC–MS-grade water; Sigma-Aldrich) and solvent B (0.05% formic acid in LC–MS-grade methanol; Sigma-Aldrich). The program, with a constant flow rate of 0.2 mL·min^−1^, was set as follows: (i) 95% solvent A/5% solvent B for 1 min 30 s; (ii) followed by 6 min in which solvent B gradually increased till 98%; (iii) continuing with 98% solvent B for 5 min; (iv) then a rapid (in 1 min) but gradual decrease returning to 95% solvent A/5% solvent B until the end of the run. A negative electrospray ionization mode was used for detection. For LC–MS/MS parameters see [52]. For all oxylipins and ABA, we used D_5_-JA to estimate the recovery rate and their *in planta* concentrations were subsequently quantified using the external standard series. For SA we used D_6_-SA to estimate the recovery rate and it was quantified using the external standard. Phytohormone amounts were expressed ng·g^−1^ FW.

#### 4.4.3. Gene Expression Analysis by Quantitative Reverse-Transcription PCR 

To determine the effect of mite-associated bacteria on defence gene expression, we performed qRT-PCRs on plant defence marker genes. Therefore, total RNA was isolated from the tomato leaf tissue that was used for phytohormone isolation, using the hot phenol method [119]. RNA integrity was checked by agarose-gel electrophoresis and a NanoDrop spectrophotometer (ND-1000; Thermo Fisher Scientific, Waltham, MA, USA) was used to assess RNA purity and quantity. Per sample, 3 μg DNase (Ambion, Austin, TX, USA)-treated RNA was used as template for reverse transcription and first strand cDNA synthesis using RevertAid H minus reverse transcriptase (Thermo Fisher Scientific). For gene expression analysis, 1 μL of 10-times diluted cDNA (i.e., the equivalent of 7.5 ng total RNA) served as template in a 20 μL qRT-PCR using the 5× HOT FIREPol EvaGreen qPCR Mix Plus (ROX) kit (Solis Biodyne, Tartu, Estonia) and the ABI 7500 real-time PCR system (Applied Biosystems, Foster City, CA, USA), according to the instructions of the manufacturers. We monitored expression of a gene involved in JA biosynthesis; *OPR3* [120], as well as JA-defence marker genes; *JIP-21* [121], *TD-2* [122], *PI-IIc* [123], SA-defence marker genes; *PR-1a* [124], *PR-P6* [124], and putative OPDA-responsive genes; *TWI-1* [125] and an uncharacterized “*GRX*” (Solyc07g053550.1). The amino acid sequences of established *Arabidopsis thaliana* ORGs [78,82,87] were used to identify their putative tomato homologs: At2g15480 (*AtUGT73B5*) for *SlTWI-1* and At1g28480 (*AtGRX480*) for tomato *GRX*. *Actin* was used as a reference gene to normalize expression data and hence correct for variance in quantity of cDNA input. Standard dilution series of selected samples were included with each qRT-PCR run to calculate primer efficiency. Amplicons generated by PCR were sequenced to verify primer specificity. Gene identifiers, primer sequences and references are listed in Table S1. The normalized expression (referred to as NE in Figure 5 and Figure 6) data were calculated by the Δ*C*_t_ method: NE = (PE_target_*^C^*^t_target^)/(PE_reference_*^C^*^t_reference^), in which PE is the primer efficiency and Ct the number of cycles to reach the cycle threshold value. 

#### 4.4.4. Statistical Analysis of the Plant Infestation Assay Data—Phytohormones, Quantitative Reverse-Transcription PCR, Feeding Damage

To test the effect of bacteria on phytohormone levels, tomato gene expression and amount of spider mite-inflicted feeding damage, we constructed LMMs using the package lme4 [116]. The respective amounts of phytohormones (ng·g^−1^ FW), normalized gene expression (NE) or total amount of feeding damage (mm^2^) for three leaflets of one plant combined were used as response variable, while “presence of bacteria” (i.e., W/S/C) was used as explanatory variable. To test the level of defences in mite-infested plants compared to non-infested control plants, in the case of phytohormones and gene expression this explanatory variable also included control plants that were not infested. Since we had four lines which were present as sublines in all bacterial “groups”, we added “line” as a random factor to the model to account for variation between the lines. The response variables were transformed using log, sqrt or 1/sqrt if applicable for meeting the assumptions of homogeneity of variance and normality of residuals required for LMM. For pairwise comparisons, we used Tukey contrasts with Holm adjustment for multiple comparisons in the multcomp package [117]. To assess how the phytohormones SA and ODPA correlated with expression levels of tomato genes (*TWI-1*, *GRX* and *OPR3*), we calculated linear correlations between phytohormone amounts and the NE level of these genes using the R package Hmisc 3.15 [126]. Further, we calculated linear correlations between SA amounts and NE levels of the SA marker genes *PR-P6* and *PR-1a*. *p*-Values of correlations were adjusted for multiple testing, using the Holm method. All analyses were performed using the statistical software R 3.0.2 [118].

## 5. Conclusions

The *T. urticae* DeLier-1 plant defence-suppressor and the Santpoort-2 defence-inducer strains harbour different bacteria. While the DeLier-1 strain harbours *Wolbachia* and *Spiroplasma* (W+S+), the Santpoort-2 strain harbours *Cardinium* and *Spiroplasma* (C+S+). In the DeLier-1 strain, the presence of *Wolbachia* was positively correlated with survival of the mites, while the presence of *Spiroplasma* did not correlate with survival. These results were not reflected in the pattern of induced plant responses. Here, the sole presence of *Spiroplasma* (and thus the absence of *Wolbachia*) was correlated with lower accumulation of OPDA and higher accumulation of SA in tomato leaflets compared to infestation of leaflets with other mite groups. Since the bacteria-correlated patterns of mite performance and plant response were not congruent, we conclude that the bacteria that we investigated are unlikely to affect plant resistance against the DeLier-1 strain of *T. urticae* on tomato plants. Rather, these bacteria may affect mite performance and plant responses independently. 

For the Santpoort-2 strain we found that the presence of *Spiroplasma* and *Cardinium* correlated negatively with feeding damage and oviposition, while it correlated positively with the induction of SA marker gene expression as well as with the rusty scar phenotype of infested leaves. This indicates a distinct and clearly visible impact of the presence of *Cardinium* and *Spiroplasma* on the mite’s host plant. Our results further suggest that the enhanced SA response that was correlated with bacterial presence may be negatively correlated with performance of the mites. However, a clear causal link between the effect of bacteria on plant responses and mite performance remains to be investigated. 

## Figures and Tables

**Table 1:** Antibiotics treatments of two strains of the spider mite *T.etranychus urticae* resulted in the (near) complete removal of their associated bacteria *Wolbachia*, *Spiroplasma* and/or *Cardinium*, as demonstrated by Illumina 16S rRNA amplicon-sequencing. The *T. urticae* DeLier-1 strain contained *Wolbachia* and *Spiroplasma* (W+S+) and tetracycline treatments either removed only *Wolbachia* or both bacteria, yielding the groups W−S+ and W−S−, respectively. The Santpoort-2 strain harboured *Cardinium* and *Spiroplasma* (C+S+), which were both removed by the tetracycline treatments, yielding the C−S− group. Each group is represented by four independent mite lines. We refer to one line in one group as a subline (e.g., line 1 in the W+S+ group). Note that mites from lines with the same number are “sister lines” which originate from the same untreated adult female. Shown are the overall total number of Illumina reads obtained per subline, as well as the total number of reads corresponding to all 11 *Wolbachia* operational taxonomic units (OTUs), all 3 *Spiroplasma* OTUs and the only *Cardinium* OTU identified. The 16S rRNA amplification was done with universal 341F and 785R primers, modified from [61], see Table S1.

Mite Strain	Group	Line	Total	*Wolbachia*	*Spiroplasma*	*Cardinium*
**DeLier-1**	**W+S+**	**1**	3164	1272	141	-
		**2**	14,303	2131	350	1
		**3**	34,118	7940	1605	2
		**4**	19,747	8546	890	-
	**W−S+**	**1**	4371	1	440	-
		**2**	16,637	-	1120	1
		**3**	27,939	6	2422	2
		**4**	8812	1	567	-
	**W−S−**	**1**	4975	3	-	-
		**2**	20,054	4	-	1
		**3**	10,241	2	-	-
		**4**	8906	1	-	-
**Santpoort-2**	**C+S+**	**5**	26,384	7	1159	7443
		**6**	18,367	5	165	3643
		**7**	48,325	108	3555	19,744
		**8**	28,966	204	1467	7904
	**C−S−**	**5**	39,276	100	1	2
		**6**	22,648	110	1	1
		**7**	30,608	66	-	3
		**8**	18,702	63	6	-

## Supplementary Materials

Supplementary materials can be found at www.mdpi.com/1422-0067/18/1/182/s1.
