# Supplementary material for: Independent Effects of a Herbivore’s Bacterial Symbionts on Its Performance and Induced Plant Defences
Source: Int J Mol Sci. 2017 Jan 18;18(1):182. doi: 10.3390/ijms18010182 (PMC5297814; doi:10.3390/ijms18010182)
Supplement: Supplementary file 1 [file ijms-18-00182-s001.pdf]

Supplementary Materials: Independent Effects of a Herbivore's Bacterial Symbionts on Its Performance and Induced Plant Defences

Heike Staudacher, Bernardus C. J. Schimmel, Mart M. Lamers, Nicky Wybouw, Astrid T. Groot and Merijn R. Kant

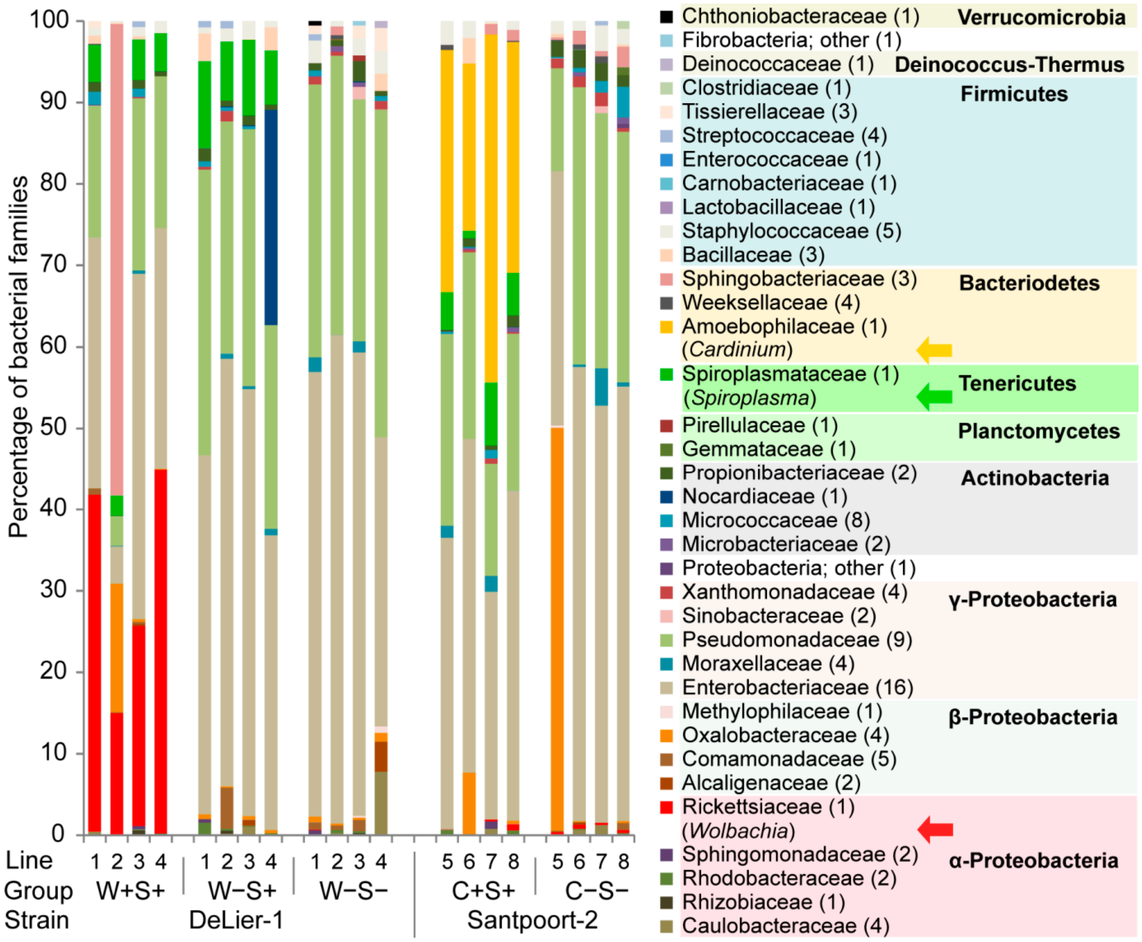

Figure S1. Bacterial community composition of two strains of the spider mite *Tetranychus urticae*, assessed with Illumina 16S rRNA amplicon-sequencing. The two strains had been treated with tetracycline and hence did (+) or did not (−) contain *Wolbachia* (W), *Spiroplasma* (S) and/or *Cardinium* (C); The DeLier-1 strain with three groups: W+S+, W−S+ and W−S−; The Santpoort-2 strain with two groups: C+S+ and C−S−; 4 lines per group. Bacterial OTUs were combined at the family level. Numbers after family names in parentheses indicate how many OTUs of one family were combined. Rare OTUs that were overall represented less than 0.5% are not shown.

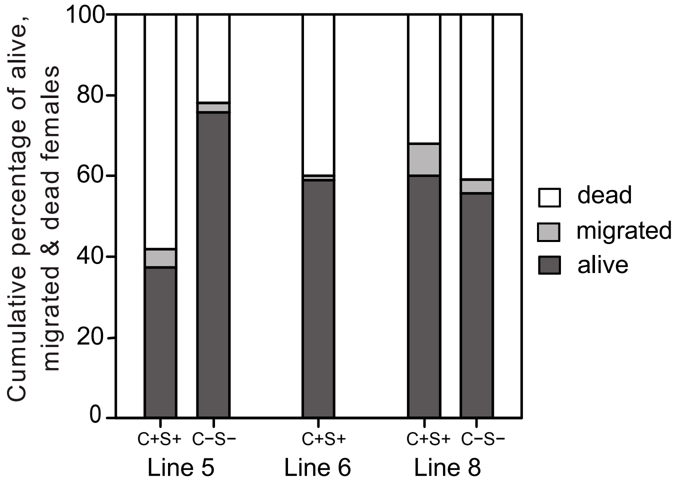

Figure S2. Survival, migration and mortality of the Santpoort-2 strain of the spider mite *T. urticae* which did (+) or did not (−) contain *Spiroplasma* and *Cardinium* after feeding on tomato (*Solanum lycopersicum*) for four days. Two lines (5 and 8) were tested with two mite groups: C+S+ and C−S−. Additionally, we tested C+S+ of line 6. C−S− of line 6 as well as both C−S− and C+S+ of line 7 went extinct before the experiment.

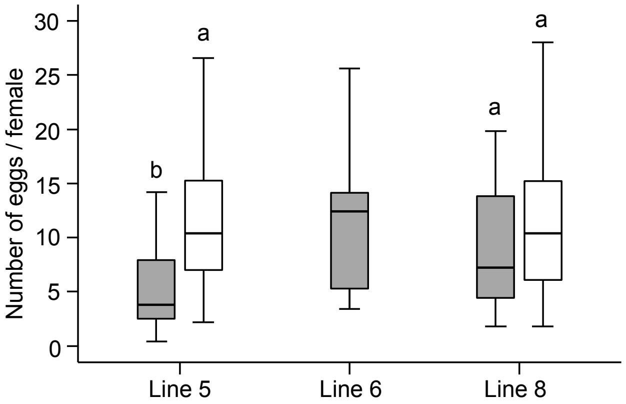

Figure S3. Reproductive performance (number of eggs produced per female in four days) of two lines (5 and 8) of the Santpoort-2 strain of the spider mite *T. urticae.* Each of the lines was subdivided into two groups which did (+) or did not (−) contain the bacteria *Cardinium* and *Spiroplasma*: C+S+ (grey) and C−S− (white). Additionally we tested C+S+ of line 6; C−S− of line 6 as well as both C−S− and C+S+ of line 7 went extinct before the experiment. Boxes span the 25–75 percentiles, horizontal lines in the boxes represent medians, whiskers span 1.5× IQR , dots represent data points outside of this range. Different letters above the boxes indicate significant differences at a level of $p \leq 0.05$ (tested per line) after applying a linear mixed model.

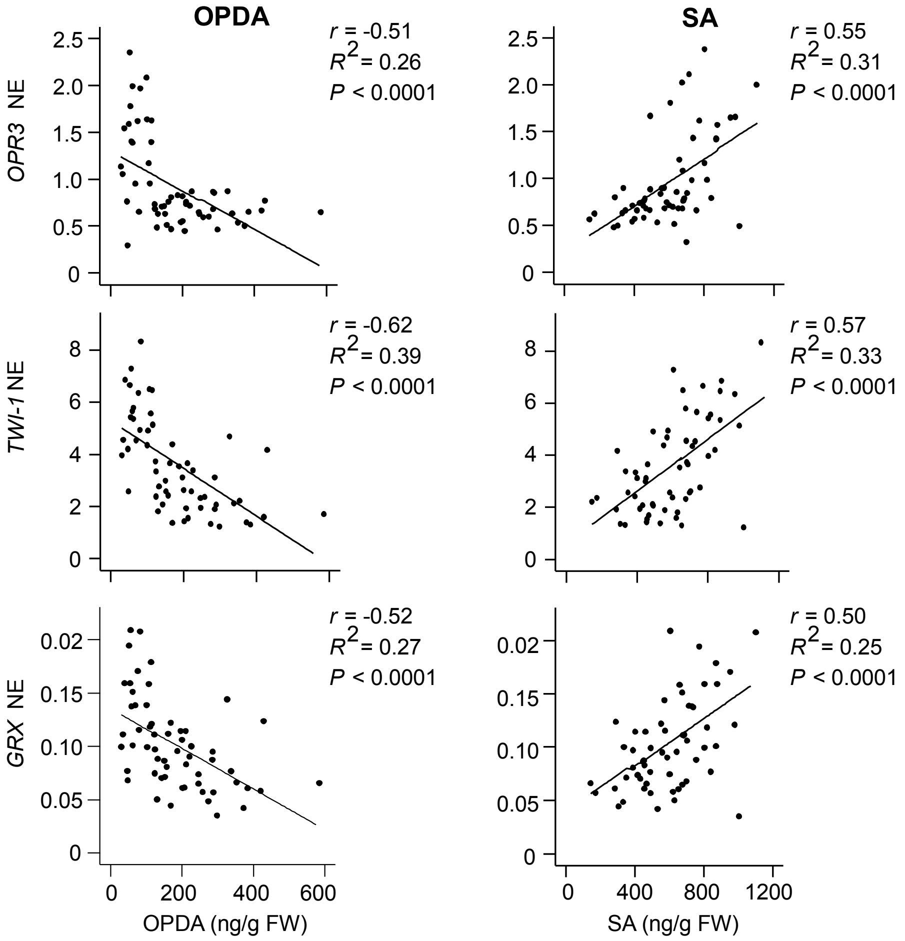

Figure S4. Correlations between phytohormone amounts of salicylic acid (SA) and 12-oxo-phytodienoic acid (OPDA) and the normalized expression levels of putative OPDA responsive genes (*OPR3*, *GRX* and *TWI-1*) measured in tomato leaflets after seven days of infestations with the Delier-1 strain of the spider mite *T. urticae*. *p*-Values were adjusted for multiple comparisons with the Holm method.

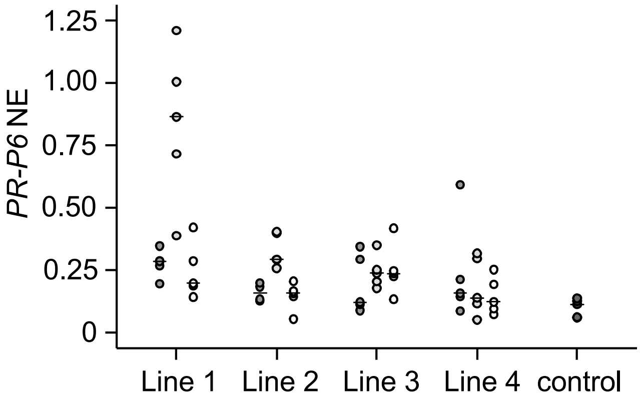

Figure S5. Normalized expression (NE) of salicylic acid defence marker gene *PR-P6* (qRT-PCR) in tomato (*S. lycopersicum*) leaflets after seven days of infestation with four lines (1, 2, 3 and 4) of the Delier-1 strain of the spider mite *T. urticae.* Each of the lines was subdivided into three groups which did (+) or did not (−) contain the bacteria *Wolbachia* and *Spiroplasma*: W+S+ (dark grey), W−S+ (light grey) and W−S− (white). Control plants were not infested (darkest grey); Circles represent individual data points, horizontal lines indicate the medians.

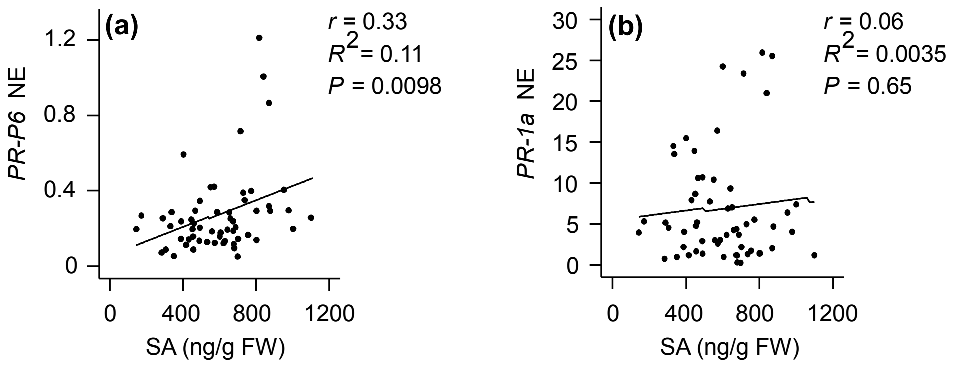

Figure S6. Correlations between phytohormone amounts of salicylic acid (SA) and the normalized expression levels of the two SA marker genes *PR-P6* and *PR-1a,* measured in tomato leaflets after seven days of infestations with the Delier-1 strain of the spider mite *T. urticae*. *p*-values were adjusted for multiple comparisons with the Holm method.

Table S1. Nucleotide sequence of primers used for PCR and qRT-PCR analysis.

Target Organism(s)	Target Gene	Name	Gene Identifier (BOGAS/ITAG2.3)	Forward Primer 5′ → 3′	Reverse Primer 5′ → 3′	References
Wolbachia pipientis	*16SrRNA*	*16S ribosomal RNA*	-	TTGTAGCCTGCTATGGTATAACT	GAATAGGTATGATTTTCATGT	[1]
Cardinium sp. (Cytophaga-Like Organism, CLO)	*16SrRNA*	*16S ribosomal RNA*	-	GCGGTGTAAAATGAGCGTG	ACCTMTTCTTAACTCAAGCCT	[2]
Spiroplasma sp.	*dnaA*	*DnaA*	-	ATTCTTCAGTAAAAATGCTTGGA	ACACATTTACTTCATGCTATTGA	[3]
Bacteria (general)	*16SrRNA*	*16S ribosomal RNA*	-	TCCTACGGGNGGCWGCAG	TGACTACHVGGGTATCTAAKCC	LGC Genomics (Berlin, Germany); modified from [4]
Tetranychus urticae	*Actin*	*Actin*	Tetur03g09480	CAGCCATGTATGTTGCCATC	AAATCACGACCAGCCAAATC	[5]
Solanum lycopersicum	*OPR3*	*OPDA Reductase 3*	Solyc07g007870.2	GATCCAGTTGTGGGATACACAG	GCCCAACAAAATCAGGTTTC	[6]
Solanum lycopersicum	*TWI-1*	*Tomato Wound-Induced 1*	Solyc01g107820.2	CATCTTACAATGGATGGGCTAC	CGAGATGATTGATCTTGGATTC	[7]
Solanum lycopersicum	*GRX*	*Glutaredoxin*	Solyc07g053550.1	ATGATGCAACAAGCACTTCC	GATGATGTCGATCAACTCTTGG	This study
Solanum lycopersicum	*JIP-21*	*Jasmonate-Inducible Protein 21*	Solyc03g098790.1	ACTCGTCCTGTGCTTTGTCC	CCCAAGAGGATTTTCGTTGA	[8]
Solanum lycopersicum	*TD-2*	*Threonine Deaminase-2*	Solyc09g008670.2	TGCCGTTAAAAATGTCACCA	ACTGGCGATGCCAAAATATC	[9]
Solanum lycopersicum	*PR-1a*	*Pathogenesis-Related Protein 1a*	Solyc09g007010.1	TGGTGGTTCATTTCTTGCAACTAC	ATCAATCCGATCCACTTATCATTTTA	[10]
Solanum lycopersicum	*PR-P6*	*Pathogenesis-Related Protein 6*	Solyc00g174340.1	GTACTGCATCTTCTTGTTTCCA	TAGATAAGTGCTTGATGTGCC	[10]
Solanum lycopersicum	*PI-IIc*	*Proteinase Inhibitor IIc*	Solyc03g020050.2	CAGGATGTACGACGTGTTGC	GAGTTTGCAACCCTCTCCTG	[11]
Solanum lycopersicum	*Actin*	*Actin*	Solyc03g078400.2	TCAGCACATTCCAGCAGATGT	AACAGACAGGACACTCGCACT	[12]

References

1. O'Neill, S.L.; Giordano, R.; Colbert, A.; Karr, T.L.; Robertson, H.M. 16S rRNA phylogenetic analysis of the bacterial endosymbionts associated with cytoplasmic incompatibility in insects. *Proc. Natl. Acad. Sci. USA* **1992**, *89*, 2699–2702.
2. Weeks, A.R.; Velten, R.; Stouthamer, R. Incidence of a new sex–ratio–distorting endosymbiotic bacterium among arthropods. *Proc. R. Soc. B Biol. Sci.* **2003**, *270*, 1857–1865.
3. Fukatsu, T.; Tsuchida, T.; Nikoh, N.; Koga, R. *Spiroplasma* symbiont of the pea aphid, *Acyrthosiphon pisum* (Insecta: Homoptera). *Appl. Environ. Microbiol.* **2001**, *67*, 1284–1291.
4. Klindworth, A.; Pruesse, E.; Schweer, T.; Peplies, J.; Quast, C.; Horn, M.; Glöckner, F.O. Evaluation of general 16S ribosomal RNA gene PCR primers for classical and next-generation sequencing-based diversity studies. *Nucleic Acids Res.* **2012**, *41*, e1.
5. Feng, H.; Wang, L.; Liu, Y.; He, L.; Li, M.; Lu, W.; Xue, C. Molecular characterization and expression of a heat shock protein gene (HSP90) from the carmine spider mite, *Tetranychus cinnabarinus* (Boisduval). *J. Insect Sci.* **2010**, *10*, 112.
6. Strassner, J.; Schaller, F.; Frick, U.B.; Howe, G.A.; Weiler, E.W.; Amrhein, N.; Macheroux, P.; Schaller, A. Characterization and cDNA-microarray expression analysis of 12-oxophytodienoate reductases reveals differential roles for octadecanoid biosynthesis in the local versus the systemic wound response. *Plant J.* **2002**, *32*, 585–601.
7. Truesdale, M.R.; Doherty, H.M.; Loake, G.J.; McPherson, M.J.; Roberts, M.; Bowles, D.J. Molecular cloning of a novel wound-induced gene from tomato: Twi1. *Plant Physiol.* **1996**, *112*, 446.
8. Lisón, P.; Rodrigo, I.; Conejero, V. A novel function for the cathepsin D inhibitor in tomato. *Plant Physiol.* **2006**, *142*, 1329–1339.
9. Chen, H.; Wilkerson, C.G.; Kuchar, J.A.; Phinney, B.S.; Howe, G.A. Jasmonate-inducible plant enzymes degrade essential amino acids in the herbivore midgut. *Proc. Natl. Acad. Sci. USA* **2005**, *102*, 19237–19242.
10. Van Kan, J.A.; Joosten, M.H.; Wagemakers, C.A.; van den Berg-Velthuis, G.C.; de Wit, P.J. Differential accumulation of mRNAs encoding extracellular and intracellular PR proteins in tomato induced by virulent and avirulent races of *Cladosporium fulvum*. *Plant Mol. Biol.* **1992**, *20*, 513–527.
11. Gadea, J.; Mayda, M.E.; Conejero, V.; Vera, P. Characterization of defense-related genes ectopically expressed in viroid-infected tomato plants. *Mol. Plant Microbe Interact.* **1996**, *9*, 409–415.
12. Consortium, T.G. The tomato genome sequence provides insights into fleshy fruit evolution. *Nature* **2012**, *485*, 635–641.
